# A Feed-Forward Circuit of Endogenous* PGC-1α* and* Estrogen Related Receptor α* Regulates the Neuronal Electron Transport Chain

**DOI:** 10.1155/2016/2405176

**Published:** 2016-03-03

**Authors:** Rachit Bakshi, Shuchi Mittal, Zhixiang Liao, Clemens R. Scherzer

**Affiliations:** ^1^Neurogenomics Lab and Harvard Parkinson Personalized Medicine Initiative, Harvard Medical School and Brigham and Women's Hospital, Cambridge, MA 02139, USA; ^2^Department of Neurology, Brigham and Women's Hospital, Boston, MA 02115, USA; ^3^Department of Neurology, Massachusetts General Hospital, Boston, MA 02114, USA; ^4^Biomarkers Program, Harvard NeuroDiscovery Center, Boston, MA 02115, USA

## Abstract

Peroxisome proliferator-activated receptor  *γ* coactivator 1*α* (*PGC-1α*) is a central regulator of cellular and mitochondrial metabolism. Cellular bioenergetics are critically important in “energy-guzzling” neurons, but the components and wiring of the transcriptional circuit through which* PGC-1α* regulates the neuronal electron transport chain have not been established. This information may be vital for restoring neuronal bioenergetics gene expression that is compromised during incipient Parkinson's neuropathology and in aging-dependent brain diseases. Here we delineate a neuronal transcriptional circuit controlled by endogenous* PGC-1α*. We show that a feed-forward circuit of endogenous neuronal* PGC-1α* and the orphan nuclear estrogen-related receptor *α* (*ERRα*) activates the nuclear-encoded mitochondrial electron transport chain.* PGC-1α* not only* trans*-activated expression of* ERRα*, but also coactivated* ERRα* target genes in complexes I, II, IV, and V of the neuronal electron transport chain via association with evolutionary conserved* ERRα* promoter binding motifs. Chemical activation of this transcriptional program induced transcription of the neuronal electron transport chain. These data highlight a neuronal transcriptional circuit regulated by* PGC-1α* that can be therapeutically targeted for Parkinson's and other neurodegenerative diseases.

## 1. Introduction

*PGC-1α* is a central regulator of cellular and mitochondrial metabolism in metabolically highly active nonneuronal cell types—brown fat cells, cardiomyocytes, and muscle cells [1].* PGC-1α* dysfunction is linked to diseased states of these cell types such as diabetes [2], cardiomyopathy [3], and sarcopenia [4].* PGC-1α* orchestrates a remodeling of cells to increase “clean energy” production [5]. It quantitatively and qualitatively increases energy production as well as the detoxifying enzymes necessary to remove the reactive oxygen species that are the byproduct of increased ATP production [1].* PGC-1α* induces mitochondrial biogenesis in response to a number of physiological clues such as exercise, cold, and fasting [1]. It remodels individual organelles by increasing levels of electron transport chain (ETC) complexes as well as ATP synthase within isolated mitochondria [4, 6].

The brain is the most energy-demanding organ [7], but the components and wiring of the transcriptional circuits through which* PGC-1α* regulates energy production in brain have not been dissected. This is in contrast to other cell types and organs for which considerable progress has been made in elucidating* PGC-1α* function [1, 4, 6, 8–13]. This information may be vital for restoring the neuronal bioenergetics that are compromised in several brain diseases, including Parkinson's (PD) [14], Huntington's (HD) [15, 16], and amyotrophic lateral sclerosis (ALS) [17].

We previously meta-analyzed laser-captured human dopamine neuron and substantia nigra transcriptomes of hundreds of individuals with Parkinson's and controls, followed by two-stage replication [14]. We found ten gene sets (i.e., groups of transcripts that encode the same biological pathway) with previously unknown associations with PD [14]. These gene sets pinpointed defects in mitochondrial electron transport, glucose utilization, and glucose sensing and indicated that these systems changes may occur already at earliest, subclinical stages of Lewy body neuropathology. Genes controlling cellular bioenergetics that are expressed in response to* PGC-1α* were underexpressed in dopaminergic neurons laser-captured from substantia nigra of motor PD patients [14]. Mechanistically, transduction with* PGC-1α* blocked mutant *α*-synuclein and rotenone toxicity in rat primary mesencephalic cultures [14]. Other laboratories showed that* PGC-1α* potently modulates dopaminergic neurodegeneration in two mouse models of PD [18–20]. The findings in sporadic PD are supported in a* PARK2*-linked, autosomal recessive variant of PD [19], where repression of* PGC-1α* by the parkin substrate PARIS contributes to neurodegeneration [19].

Here we set out to clarify a specific, open question: the transcriptional circuit through which endogenous* PGC-1α* regulates the neuronal electron transport chain in neuronal cells and brain. Our data indicate that endogenous* PGC-1α* and estrogen-related receptor *α* (*ERRα*) coactivate the nuclear-encoded electron transport chain in neuronal cells through a feed-forward loop. This transcriptional network can now be further defined and therapeutically exploited as chemical activation induced a pervasive increase in endogenous neuronal electron transport chain gene expression.

## 2. Materials and Methods

### 2.1. Mouse Brains

Snap-frozen whole brain tissue from* PGC-1α* KO mice, originally characterized by Dr. Bruce Spiegelman (Dana-Farber Cancer Institute, Harvard Medical School), were obtained from Jackson Laboratory (stock number 008597). All animal experiments were carried out in accordance with the National Institutes of Health Guide for the Care and Use of Laboratory Animals and were approved by the local animal care committee.

### 2.2. Cell Culture

SK-N-MC neuroblastoma cells were maintained in Dulbecco's modified Eagle's medium supplemented with 10% (v/v) FCS. All cells were cultured in the presence of 100 U/mL penicillin and 100 *μ*g/mL of streptomycin sulfate in 5% CO_2_ at 37°C.

### 2.3. Transfections and Adenoviral Transductions

Low passage SK-N-MC cells were plated at 8 × 10^5^ cells/well in a 6-well plate the day before transfection in media lacking antibiotics. Routinely, cells were transfected with a total of 1 to 5 *μ*g of plasmid DNA using Lipofectamine 2000 (Invitrogen) following the manufacturer's instructions. For adenoviral transductions, SK-N-MC cultures were transduced with adenovirus encoding* PGC-1α* or LacZ (50 MOI) for 24 hours as described elsewhere [12]. Cells were harvested after 48 hours of treatment.

### 2.4. RNA Isolation and Quantitative Real-Time PCR

RNA was extracted from SK-N-MC cells or snap-frozen brain tissue samples by TRIzol (GIBCO/BRL) extraction similar to what we describe in [12]. RNA quality was determined by spectrophotometry and by visual inspection of electropherograms using the RNA 6000 NanoChip Kit on the Agilent 2100 Bioanalyzer (Agilent Technologies). For quantitative gene expression analysis in human biospecimens, TaqMan Assay-on-demand primers and probes (Applied Biosystems) were used. Amplification products were analyzed for specificity by agarose gel electrophoresis. To detect* PGC-1α* mRNA, we have used TaqMan probe Hs01016719_m1, which does not differentiate between various* PGC-1α* isoforms. The comparative threshold cycle method was used for analysis. Glyceraldehyde-3-phosphate dehydrogenase (*GAPDH*) and* RPL13* ribosomal RNA were used as RNA loading controls. Equal amplification efficiencies were confirmed for target and reference genes.

### 2.5. siRNA Transfection

Low passage SK-N-MC cells were seeded into 6-well dishes at 40% confluency. The required amount of target siRNA (Invitrogen) and 9 *μ*L of Lipofectamine RNAi MAX (Invitrogen) were each diluted into a final volume of 250 *μ*L in Opti-MEM (GIBCO), then combined, gently mixed, and incubated at room temperature for 25 min. 500 *μ*L of this transfection solution was overlaid onto cells at a final concentration of 80 nM siRNA. Transfection of SK-N-MC cells with RNAi Negative Control (Dharmacon, with no significant homology to any known gene sequences from mouse, rat, or human) served as a negative control. After 48 hr incubation at 37°C in the presence of 5% CO_2_, cells were lysed by TRIzol reagent, and total RNA was isolated by chloroform/isopropanol precipitation. To detect* PGC-1α* protein levels by Western blot analysis we used a rabbit polyclonal antibody (H300, Santa Cruz, CA, USA).

### 2.6. Quantitative Chromatin Immunoprecipitation Analysis

Chromatin immunoprecipitation assays (ChIPs) were performed in asynchronously growing SK-N-MC cells transfected with the myc-*PGC-1α* construct or the empty vector. Cross-linking was carried out with 1% formaldehyde for 10 min at room temperature. Cross-linking was subsequently quenched by adding glycine to a final concentration of 250 mM for 10 min. Cells were collected and washed twice with PBS and then resuspended in 2.5 mL of lysis buffer (150 mM NaCl, 50 mMTris-HCl pH 8.0, 1% NP-40, 25 *μ*M MG-132, and 1x Complete® Protease inhibitor cocktail). After 10 min on ice, cells were sonicated to obtain DNA fragments of ~500 bp as determined by agarose gel electrophoresis with ethidium bromide staining. Protein-DNA complexes were isolated by centrifugation at 15,000 rpm for 20 min. Supernatants with protein-DNA complexes were incubated for 16 hrs with rabbit polyclonal antibody directed against* PGC-1α*. Normal rabbit IgG was used as a control. Antibody-protein-DNA complexes were further incubated with 100 *μ*L of magnetic DYNA beads (Invitrogen) to isolate antibody bound fractions of chromatin. Immunocomplexes were washed with the following buffers: low salt (20 mM Tris-Cl, pH 8.1, 150 mM NaCl, 1% Triton X-100, 2 mM EDTA, and 1x complete protease inhibitor), high salt (20 mM Tris-Cl, pH 8.1, 500 mM NaCl, 1% Triton X-100, and 2 mM EDTA), LiCl (10 mM Tris-Cl, pH 8.1, 250 mM LiCl, 1% deoxycholate, 1% NP-40, and 1 mM EDTA), and twice in TE (10 mM Tris-Cl, pH 8.1, and 1 mM EDTA). Protein-DNA complexes were eluted in 1% SDS and 100 mM NaHCO_3_. Cross-links of pulldown fractions and inputs (2% of total IP fraction) were reversed by overnight incubation in elution buffer and 0.2 M NaCl. DNA was then extracted, purified, precipitated, and resuspended in TE for qPCR. Immunoprecipitated DNA was analyzed by real-time PCR as previously described. The primer sequences are available in supplement. The dissociation curves showed that PCRs yielded single products. Samples from three or more independent immunoprecipitation assays were analyzed.

### 2.7. Statistical Analysis

Values were expressed as mean ± standard error of the mean (SEM). Differences between groups were examined for statistical significance using one-way ANOVA or two-tailed Student's *t*-tests, using GraphPad Prism 5 software. A *P* value less than 0.05 denoted the presence of a statistically significant difference.

## 3. Results

### 3.1. Endogenous* PGC-1α* Regulates Nuclear-Encoded Electron Transport Chain Genes in Neuronal Cells and in Brain

**Figure 1:**
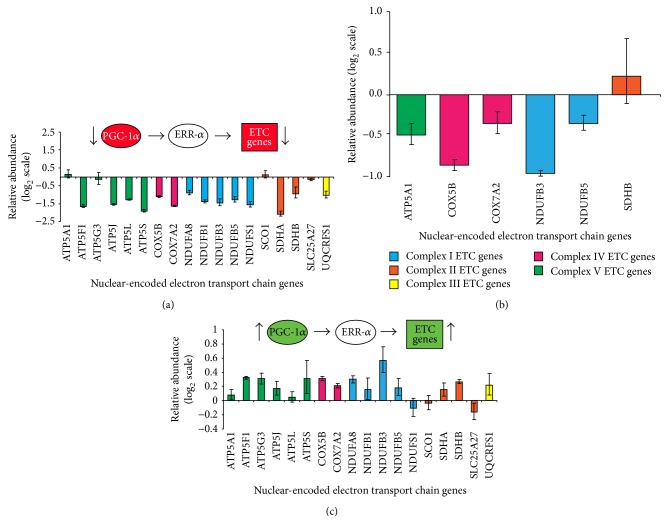
Endogenous* PGC-1α* regulates the nuclear-encoded electron transport chain genes in neuronal cells and in mice. (a) Silencing of endogenous* PGC-1α* repressed the expression of nuclear-encoded electron transport chain genes representing complexes I, II, III, IV, and V of the electron transport chain in SK-N-MC cells by quantitative PCR analysis (note log_2_ scale). Model circuit and observed effects in circuit components are shown (top). Subunits of complexes I to V of the electron transport chain are color-coded in panels (a)–(c) in accordance with the color legend shown in (b). Means ± SEM are shown (*N* = 3 for each treatment). The ribosomal gene* RPL13* was used to control for input RNA. (b) Expression of representative electron transport chain genes was similarly reduced in brain of* PGC-1α* null mice [21] compared to age- and sex-matched wild-type littermates (*N* = 3). The ribosomal gene* RPL13* was used to control for input RNA. (c) Transduction with adenovirus carrying* PGC-1α trans*-activated the expression of endogenous genes encoding nuclear subunits of complexes I, II, IV, and V of the mitochondrial respiratory chain in SK-N-MC cells compared controls transduced with the LacZ gene. Model circuit and observed effects in circuit components are shown (top).

To determine whether endogenous* PGC-1α* systematically regulates the expression of the endogenous, neuronal electron transport chain, we silenced native* PGC-1α* using small interfering RNA (siRNA) in dopaminergic SK-N-MC neuroblastoma cells. Transfection with 100 nM* PGC-1α* siRNA reliably knocked down* PGC-1α* mRNA abundance by 80% compared to cells transfected with negative control siRNA (Supplementary Figure S1, in Supplementary Material available online at http://dx.doi.org/10.1155/2016/2405176). Similar results were obtained when* UBC* instead of the ribosomal gene* RPL13* was used to control for RNA loading. Silencing of endogenous* PGC-1α* repressed the relative abundance of 14 of 18 nuclear-encoded electron transport chain genes (ETC) analyzed chosen to representing complexes I, II, III, IV, and V of the electron transport chain with *P* values below 0.05 (Figure 1(a)). Importantly, similar results were observed in brain of* PGC-1α* null mice [21]. Expression of 5 out of 6 ETC subunits probed was significantly decreased in* PGC-1α* knockout mice (Figure 1(b)) compared to age- and sex-matched wild-type littermates (*N* = 3) with *P* values below 0.05.

Conversely, we previously showed that transduction with adenovirus carrying* PGC-1α* (but not transduction with the control LacZ gene)* trans*-activated the expression of endogenous genes encoding nuclear subunits of complexes I, II, IV, and V of the mitochondrial respiratory chain in primary rat midbrain cultures [14]. We independently confirm this here in an additional cell line, SK-N-MC cells (Figure 1(c)). In the catecholaminergic SK-N-MC cells, 15 of 18 ETC genes analyzed were overexpressed in response to transduction with* PGC-1α* (Figure 1(c)). Collectively, these data show that endogenous* PGC-1α* regulates electron transport chain gene expression in neuronal cells and in brain.

### 3.2. The Orphan Nuclear Estrogen-Related Receptor *α* (*ERRα*) Is an Early Target of Endogenous, Neuronal* PGC-1α*

**Figure 2:**
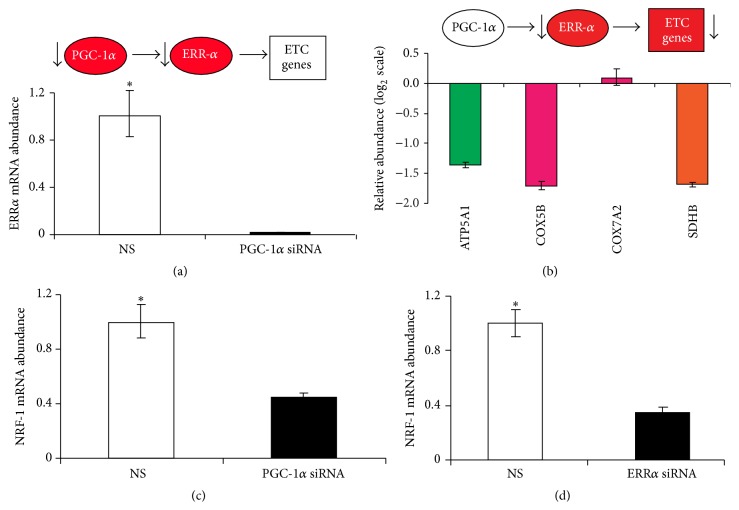
The orphan nuclear estrogen-related receptor *α* (*ERRα*) is an early target of endogenous, neuronal* PGC-1α*. (a) Silencing of neuronal* PGC-1α* repressed the expression of endogenous* ERRα* by more than 90%, respectively, compared to controls transfected with scrambled siRNAs (NS). Model circuit and observed effects in circuit components are shown. (b) Silencing of* ERRα* largely recapitulated the reduction in electron transport chain gene expression observed in response to* PGC-1α*-silencing (note log_2_ scale).* NRF1* gene expression was downregulated by more than 50% by silencing* PGC-1α* (c) or* ERRα* (d). The ribosomal gene* RPL13* was used as control for input RNA. Mean ± SEM shown (*N* = 3 for each set). *∗* denotes *P* value ≤ 0.05.

*ERRα* was identified on the basis of its sequence similarity to classical, hormone-regulated steroid receptors [23]. It recognizes similar DNA motifs as the estrogen receptors but does not bind naturally secreted estrogens in animals [24]. However,* PGC-1α* is a peptide ligand for* ERRα* in nonneuronal cells [13]. There,* PGC-1α* induces the expression of* ERRα* and potently converts* ERRα* from a factor with little or no transcriptional activity to a potent regulator of gene expression via interaction with leucine-rich motifs in the* PGC-1α* peptide [13]. To determine whether* PGC-1α* similarly exerts its regulatory control on the* neuronal *electron transport chain genes in coordination with endogenous* ERRα*, we silenced endogenous* PGC-1α* in SK-N-MC cells. Knockdown of* PGC-1α* dramatically repressed endogenous* ERRα* expression (Figure 2(a)) by more than 90% and also repressed the late target gene nuclear respiratory factor-1 (*NRF1)* by more than 50% (Figure 2(c)) compared to controls transfected with scrambled siRNAs. To further delineate the underlying transcriptional program, we then silenced endogenous* ERRα* (Supplementary Figure S2). Silencing* ERRα* not only repressed the* NRF1* gene expression (Figure 2(d)) but also recapitulated the reduction in electron transport chain gene expression observed in response to* PGC-1α*-silencing (with the exception of* COX7A2* expression) (Figure 2(b)). This is consistent with previous studies in nonneuronal cells, that is, murine myoblasts [8] and human osteosarcoma cells [13].

Collectively, these data suggest that in neuronal cells, endogenous* PGC-1α* is a potent transcriptional coactivator of the early target gene* ERRα* and that both endogenous* PGC-1α* and* ERRα* activity modulate the late target gene* NRF1 *and the expression of most components of the human neuronal electron transport chain.

### 3.3. *PGC-1α* Physically Associates with Evolutionary Conserved* ERRα* Binding Motifs in the Promoters of Neuronal Electron Transport Chain Genes That Are Dysregulated in Parkinson's Disease

**Figure 3:**
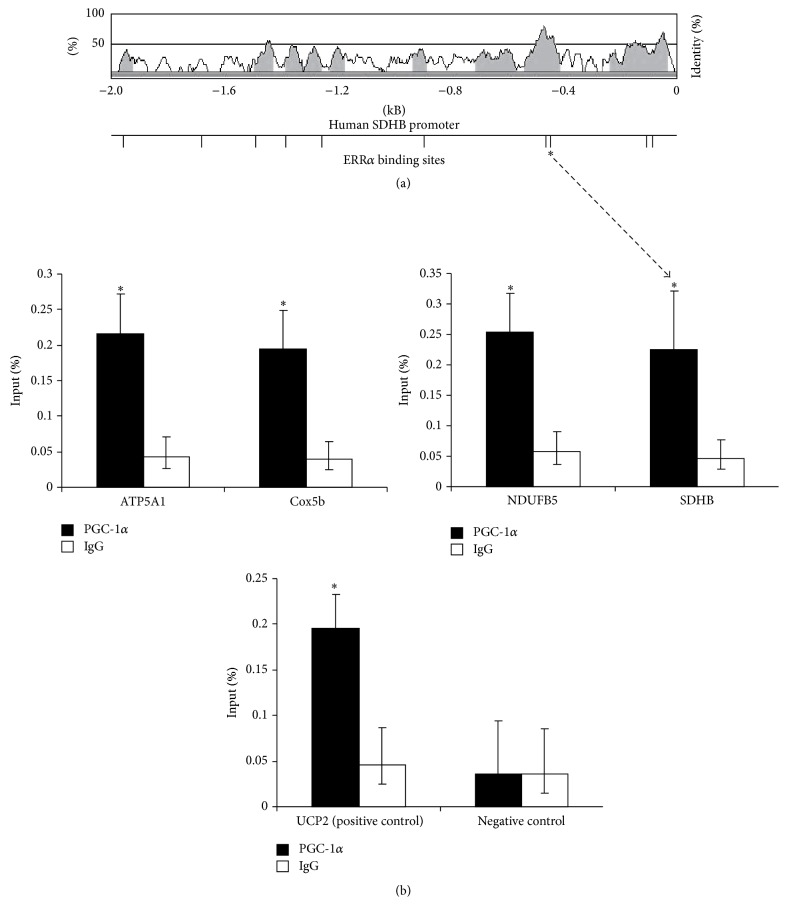
*PGC-1α* physically associates with evolutionary conserved* ERRα* binding motifs in the promoters of neuronal electron transport chain genes that are dysregulated in Parkinson's disease. (a) The VISTA plot of a 2-kb promoter region of the* SDHB* gene is shown with percentage identity of the human and mouse sequences. Small vertical bars indicate the location of conserved predicted ERRE binding motifs and the asterisk indicates the binding motif assayed by quantitative chromatin immunoprecipitation. (b) Quantitative chromatin immunoprecipitation (qChIP) analyses in SK-N-MC neuroblastoma cells transfected with a myc-tagged* PGC-1α* plasmid construct were performed. Promoter fragments for* ATP5A1 *(complex V),* COX5B *(complex IV),* NDUFB5* (complex I), and* SDHB* (complex II) were specifically enriched in the IP fraction of* PGC-1α* compared to IgG control indicating* PGC-1α* occupancy of the conserved ERRE motifs.* UCP-2*, a known transcriptional target of* PGC-1α*  [22], was used as positive control. No* PGC-1α* occupancy was seen in intergenic regions lacking a predicted* ERRα* binding site that was included as a negative control. Quantitative PCR data were normalized to genomic DNA and visualized as percent input. *∗* denotes *P* value ≤ 0.05.

Transcriptional coregulators like* PGC-1α* exert their function through transcriptional complexes that occupy the promoters of distinct target genes. Transcription factors direct these complexes (including the transcriptional coregulator) to specific target sequences. The transcription factor* ERRα* occupies a nine-nucleotide extended half-site sequence with the consensus TNAAGGTCA, referred to as* ERRα* response element (ERRE) [25, 26]. These* ERRα* binding motifs are evolutionary conserved and enriched in electron transport chain genes (Figure 3(a) and Supplementary Figure S3) [8, 27]. In order to evaluate whether* PGC-1α* regulation of ETC genes is the result of an interaction of its transcriptional complex with these evolutionarily conserved* ERRα* binding motifs, we performed quantitative chromatin immunoprecipitation (ChIP) analyses in SK-N-MC neuroblastoma cells overexpressing* PGC-1α* protein.

We evaluated* PGC-1α* cooccupancy of conserved* ERRα* in the promoters of electron transport chain genes that are underexpressed in laser-captured nigral dopamine neurons of patients with symptomatic PD neuropathology as well as in individuals with incipient, subclinical PD neuropathology [14]. One gene representative for each of complexes I, II, IV, and V of the electron transport chain was investigated.* ATP5A1 *(complex V),* COX5B* (complex IV),* NDUFB5* (complex I), and* SDHB* (complex II) were evaluated. Promoter fragments were specifically enriched in the IP fraction of* PGC-1α* compared to IgG control indicating* PGC-1α* occupancy of the conserved ERRE motifs (Figure 3(b)).* UCP-2*, a known transcriptional target of* PGC-1α*  [22], was used as positive control. No* PGC-1α* occupancy was seen in intergenic regions lacking a predicted* ERRα* binding site that were included as negative controls (Figure 3(b)).

These results indicate that* PGC-1α* not only* trans*-activates expression of the transcription factor* ERRα* but also coactivates its target genes in the neuronal electron transport chain via occupancy of conserved* ERRα* binding motifs in their promoters.

### 3.4. The Endogenous* PGC-1α* and* ERRα*-Regulated Feed-Forward Circuit Can Be Targeted through Systems Pharmacology

Pioglitazone, a thiazolidinedione approved for the treatment of diabetes, is a synthetic ligand for Peroxisome proliferator-activated receptor *γ* (*PPARγ*) and to a lesser extent* PPARα* [28].* PPARγ trans*-activates* PGC-1α* thereby activating mitochondrial biogenesis in human subcutaneous tissue [29]. Importantly, for Parkinson's disease [30], treatment with pioglitazone or with related thiazolidinediones is protective in multiple animal models of PD [31–33]. 1-Methyl-4-phenyl-1,2,3,6-tetrahydropyridine (MPTP) and rotenone are linked to parkinsonism in humans and rodents. Thiazolidinediones strongly suppress MPTP-induced-loss of tyrosine hydroxylase-positive cells in the substantia nigra pars compacta [31, 32] as well as motor and olfactory dysfunctions in animal models [32]. Pioglitazone also suppressed rotenone-induced reduction in striatal dopamine levels and locomotor activity in rats [34].

**Figure 4:**
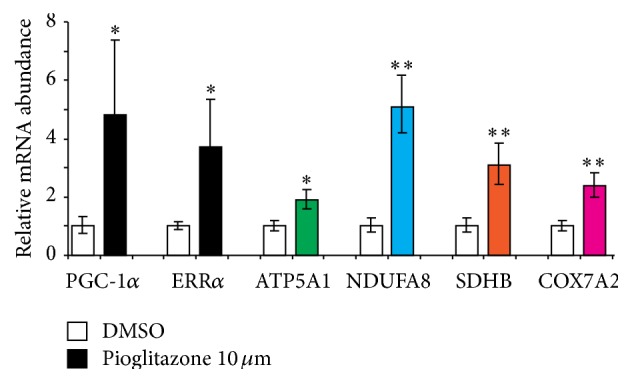
Endogenous* PGC-1α* is a target for drugs designed to restore electron transport chain expression. Treatment with pioglitazone (concentration of 10 *μ*M, 48 hours of treatment) pervasively activated the* PGC-1α*-*ERRα* circuit. It induced a statistically significant 5-fold increase in expression of endogenous* PGC-1α*, a significant 2-3-fold increase in endogenous* ERRα*, and a resulting significant 2–5-fold-*trans*-activation of their endogenous electron transport chain target genes compared to cells treated with vehicle alone. Mean ± SEM shown (*N* = 10 for each treatment). *∗* denotes *P* value ≤ 0.05. *∗∗* denotes *P* value ≤ 0.005.

To test the idea that the* PGC-1α* and* ERRα*-regulated feed-forward circuit can be exploited as a target system for therapeutics, we evaluated the endogenous transcriptional response to pioglitazone treatment in neuronal cells. A dose response curve with increasing concentrations of pioglitazone was performed (Supplementary Figure S4). At a concentration of 10 *μ*M, 48-hour treatment with pioglitazone pervasively activated the* PGC-1α*-*ERRα* circuit (Figure 4). It induced a statistically significant 5-fold increase in expression of endogenous* PGC-1α*, a significant 2-3 fold increase in endogenous* ERRα*, and a correlated, significant 2–5-fold-*trans*-activation of their electron transport chain target genes (Figure 4). These data confirm that the* PGC-1α* and* ERRα*-regulated feed-forward circuit is druggable for early intervention in PD and other brain diseases.

## 4. Discussion

Cellular bioenergetics are particularly important in “energy-guzzling” neurons, but the role of* PGC-1α* in regulating the neuronal electron transport chain has not previously been clarified. In this study we delineate a previously unconfirmed neuronal transcriptional circuit controlled by endogenous* PGC-1α*. By combining gene silencing and gene expression with quantitative chromatin immunoprecipitation analysis in neuronal cells and mouse brain, and taken together with our previous studies in primary mesencephalic cultures [14], we show evidence for a feed-forward circuit of endogenous neuronal* PGC-1α* and* ERRα* that activates the nuclear-encoded mitochondrial electron transport chain via occupancy of evolutionary conserved* ERRα* motifs.* PGC-1α*-induced ETC gene expression has been previously linked to mitochondrial respiration [35]. In muscle cells, for example,* PGC-1α*-induced ETC gene expression results in increased mitochondrial respiration [35].

Mitochondrial dysfunction is impaired in common and rare neuronal diseases. Recent studies have shown that genes involved in the nuclear-encoded electron transport chain exhibit reduced expression in dopamine neurons and substantia nigra of humans with symptomatic and subclinical Parkinson's neuropathology. Systems biology analysis of human brains revealed a pervasive expression defect of* PGC-1α*-linked bioenergetics genes in laser-captured dopamine neurons of Parkinson's patients and substantia nigra of individuals with subclinical, brainstem-predominant Lewy body neuropathology [14] that likely represent preclinical PD [36]. These findings were replicated in an independent population [37]. The gene sets identified pinpointed defects in mitochondrial electron transport, glucose utilization, and glucose sensing early in the disease course [14]. Conversely, activating the* PGC-1α*-regulated program ameliorated mutant *α*-synuclein- and rotenone-induced loss of dopamine neurons in primary midbrain cultures [14]. In mouse models of PD, the* PGC-1α* transgene suppressed MPTP-induced dopaminergic neurodegeneration [18]. Conversely, deletion of* PGC-1α* dramatically enhanced MPTP-induced degeneration of nigral dopamine neurons in a mouse model of PD [20]. In mice carrying mutant* PARK2*-linked familial PD repression of* PGC-1α* by the parkin substrate PARIS contributes to neurodegeneration, while increased* PGC-1α* expression suppressed mutant parkin-induced neurodegeneration [19]. In an isogenic human induced Pluripotent Stem Cell model of Parkinson's* PGC-1α* suppressed cell loss in response to environmental toxins and mutant *α*-synuclein [38]. In short, evidence in human brain and in multiple cellular, human stem cell, genetic and toxic animal models of PD link* PGC-1α*-regulated programs to an onset mechanism of Parkinson's. Beyond Parkinson's there are clues to suggest that a* PGC-1α-*regulated transcriptional program is more generally involved in aging-related diseases such as ALS and HD [16, 39]. Mildly increased* PGC-1α* expression in skeletal muscle protects from sarcopenia during aging [4].

Is this pathway a tractable target for gene therapy? In mice, both too little and too much* PGC-1α* are detrimental.* PGC-1α* knockout leads to cardiomyopathy [12], but forced overexpression of* PGC-1α* at supraphysiologic levels induces uncontrolled mitochondrial proliferation and cardiomyopathy [12]. Analogously, adenoassociated virus- (AAV-) mediated overexpression of* PGC-1α* in the substantia nigra induces a loss of dopaminergic markers and enhances nigral vulnerability [40, 41].

Chemically restoring the activity of the endogenous* PGC-1α*-regulated circuit (i.e., reduced in Parkinson's neuropathology) back to normal may be a more advantageous strategy for early intervention in incipient PD than forced overexpression of exogenous* PGC-1α*. This could be accomplished through small molecule drugs that modulate any of the switches in the neuronal circuit we here delineated.* PGC-1α* expression can be activated through molecules acting upstream of the* PGC-1α* gene such a glitazones. For example, pioglitazone confers neuroprotection in mouse models of PD [32] and activates the entire neuronal* PGC-1α-ERRα*-regulated feed-forward circuit in neuronal cells through activation of the nuclear receptor* PPARγ,* the transcription factor of* PGC-1α*. Because* PPARγ* regulates numerous transcriptional cascades in addition to the* PGC-1α*-regulated circuit, this approach carries the risk of side effects through broad activation of unwanted programs. Moreover, initiation of treatment during earliest, preclinical disease stages might be necessary to achieve meaningful effects. In patients with clinically manifest PD (indicating advanced underlying Lewy body neuropathology and substantial loss of dopamine neurons), no efficacy was found for pioglitazone in slowing disease progression in a clinical trial [42]. However, a large, recent epidemiologic study suggested a beneficial effect for glitazones such as pioglitazone in reducing risk of PD in neurologically normal individuals with diabetes [43]. This study found an incidence rate of PD in the glitazone-exposed group of 6.4 per 10,000 patient years compared with 8.8 per 10,000 patient years in those prescribed other antidiabetic treatments [43].* ERRα* is another switch in the circuit that could be targeted. We show that endogenous* PGC-1α* regulates neuronal* ERRα* transcription (Figure 2) and that silencing neuronal* ERRα* recapitulates the effect of* PGC-1α* knockdown on endogenous electron transport chain expression (Figure 2).* ERRα* may be both sufficient and necessary for mediating the action of* PGC-1α* on mitochondrial biogenesis as in muscle cells induction of mitochondrial biogenesis by* PGC-1α* was largely suppressed when* ERRα* was inhibited [8]. Targeting* ERRα* directly with small molecules is an attractive strategy for drug development, although the ligand-binding pocket is small [44]. Phytoestrogens activate* ERRα* [45] and a synthetic compound that inhibits* ERRα* has been reported [8, 45]. There is also precedent for a promising third strategy, targeting the* ERRα-PGC-1α* interaction with small molecules [8, 46].

These data clarify a transcriptional network regulated by neuronal* PGC-1α* that now can be therapeutically targeted for common neurodegenerative diseases. Novel chemical modulators tailored to this circuit together with a transformed clinical trial paradigm directed at individuals with earliest, preclinical stages of neuropathology will be positioned to modify neuronal bioenergetics defects and potentially achieve substantial clinical benefits for patients with neurodegenerative disease.

## Supplementary Material

The Supplementary Material includes Supplementary Figures and a Supplementary Table.
