# Supplementary material for: A Feed-Forward Circuit of Endogenous PGC-1α and Estrogen Related Receptor α Regulates the Neuronal Electron Transport Chain
Source: Parkinsons Dis. 2016 Mar 3;2016:2405176. doi: 10.1155/2016/2405176 (PMC4818948; doi:10.1155/2016/2405176)
Supplement: Supplementary file 1 — The Supplementary Material includes Supplementary Figures and a Supplementary Table. [file 2405176.f1.pdf]

Supplementary Material

Table 1: Primers used for qChIP

Gene product	Forward primer	Reverse primer
**ATP5A1**	CCCCGGTTATCAGGATTTTT	CGGGGCAATCTGAATTTTTA
**COX5B**	CTCAACTCCAGGGACCAAAA	AGCTCTGCCTCTTTCTGCAC
**NDUFB5**	TCCTACAAAGAAGGGCCAAA	ATGAGGACAAAGGCAGGATG
**SDHB**	AGGGACCCGGATAGGATG	CTTCCCCCGTGACCTTCT
**UCP2**	GCAGGCCTTTGCATCTGTTCT	TAGCTTTTGCGCTGAGCTCTG
**Negative**	TGCCAAAGCCTAGGGGAA	ATGGTTGCCACTGGGGATC

Figure Legends

Figure S1. (A) Quantitative PCR shows a dose-dependent reduction of *PGC-1α* mRNA abundance after transfection with 50–150 nM *PGC-1α* siRNA. (B) Transfection with 80 nM *PGC-1α* siRNA levels is sufficient to reliably knock down *PGC-1α* mRNA abundance to 20% of the abundance in cells transfected with negative control siRNA. (C) *PGC-1α* protein levels are substantially reduced after transfection with 80 nM *PGC-1α* siRNA by western blot analysis compared to negative control transfected and cells.

Figure S2. Transfection with ERRα siRNA reduced the ERRα mRNA abundance to 20% of the abundance in cells transfected with negative control siRNA (n=4).

Figure S3. VISTA plot of a 2kb region of the promoter showing percentage identity of the human and mouse sequences. Underlined sequence represents the ChIP primer location spanning a putative ERRα binding site.

Figure S4. Dose response curve correlating pioglitazone at three concentrations and relative abundance of PGC-*1α* mRNA.

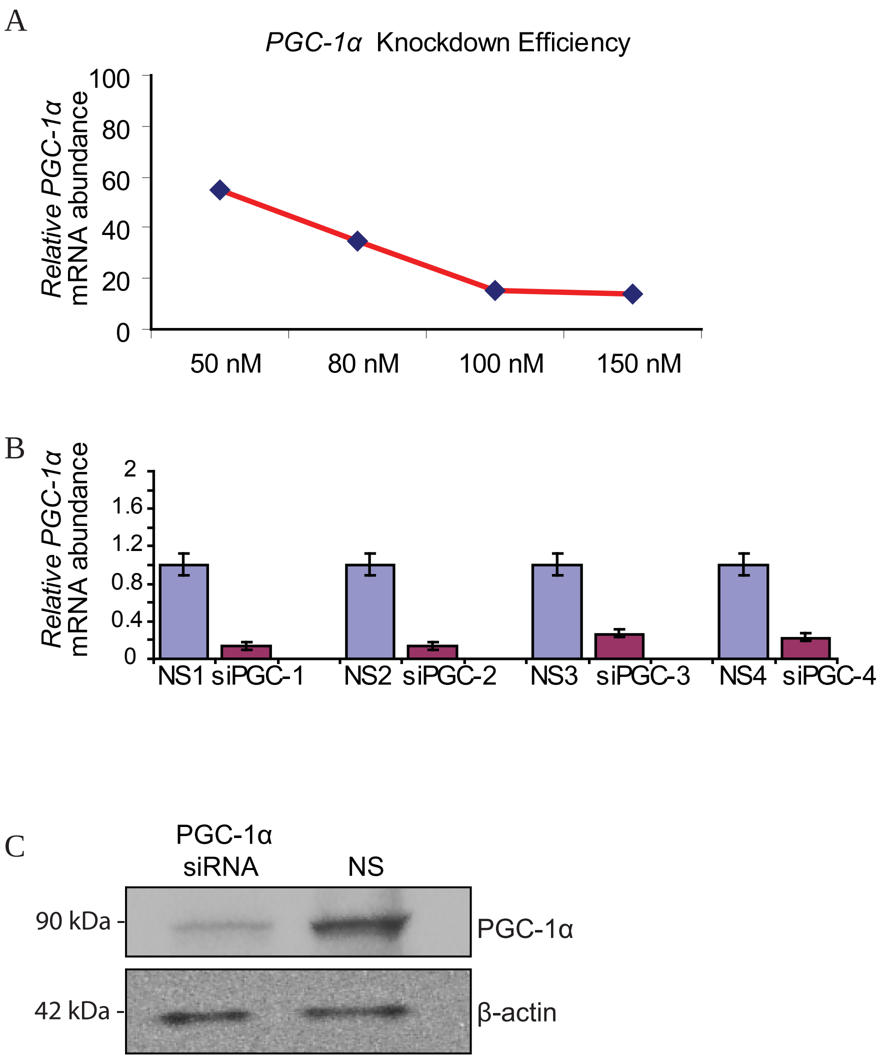
A
PGC-1α Knockdown Efficiency
Relative PGC-1α mRNA abundance
100
80
60
40
20
0
50 nM
80 nM
100 nM
150 nM
B
Relative PGC-1α mRNA abundance
2
1.6
1.2
0.8
0.4
0
NS1 siPGC-1
NS2 siPGC-2
NS3 siPGC-3
NS4 siPGC-4
C
PGC-1α siRNA
NS
90 kDa
42 kDa
PGC-1α
β-actin

Figure S1

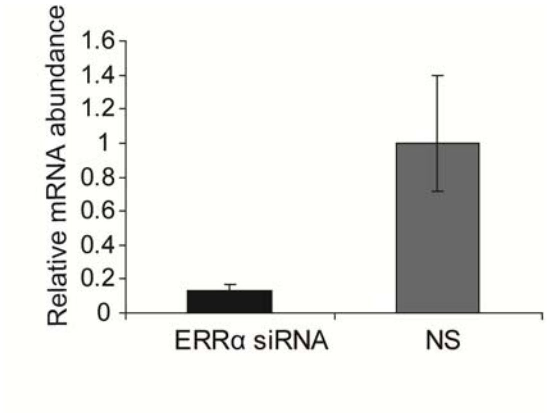

Figure S2

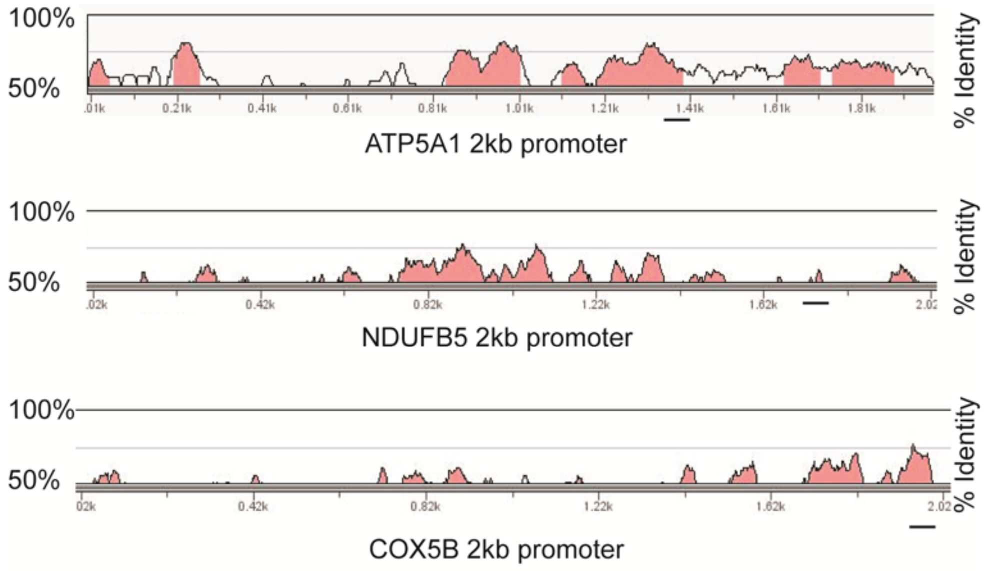

100%
50%
% Identity
ATP5A1 2kb promoter
100%
50%
% Identity
NDUFB5 2kb promoter
100%
50%
% Identity
COX5B 2kb promoter

Figure S3

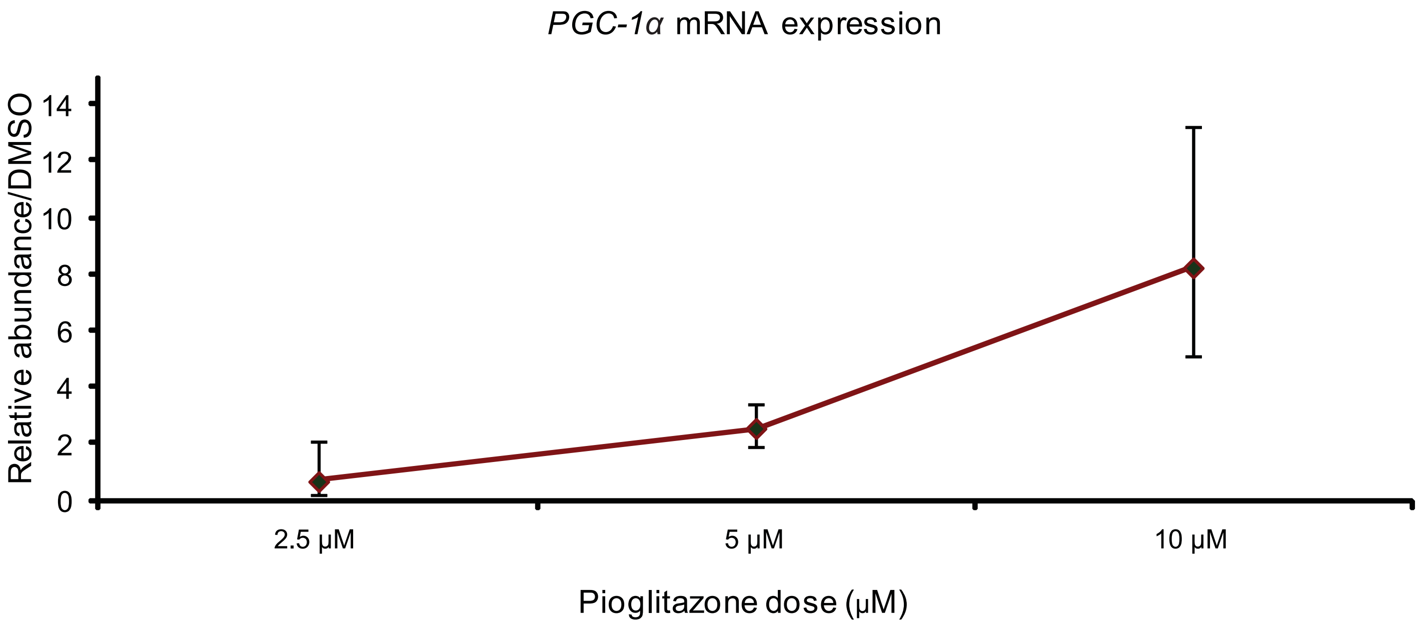

PGC-1α mRNA expression
Relative abundance/DMSO
0
2
4
6
8
10
12
14
2.5 µM
5 µM
10 µM
Pioglitazone dose (µM)

Figure S4
